# Mechanisms Underlying the Prevention and Treatment of Cholelithiasis Using Traditional Chinese Medicine

**DOI:** 10.1155/2019/2536452

**Published:** 2019-06-17

**Authors:** Qiliang Chen, Yuanyuan Zhang, Shunan Li, Shujiao Chen, Xuejuan Lin, Candong Li, Tetsuya Asakawa

**Affiliations:** ^1^Research Base of Traditional Chinese Medicine Syndrome, Fujian University of Traditional Chinese Medicine, Fuzhou 350122, China; ^2^Department of Neurosurgery, Hamamatsu University School of Medicine, Handayama, Hamamatsu-City, Shizuoka, Japan

## Abstract

Cholelithiasis is a major public health concern that necessitates highly effective, feasible, and recurrence-preventing therapies. Currently available surgical treatments and medications cannot effectively avoid the recurrence of cholelithiasis. Hence, several Chinese herbal compounds (CHCs) are considered for the treatment of cholelithiasis, considering that they can effectively discharge gallstones and prevent the recurrence of such condition. In the present narrative review, we aim to summarize the underlying mechanisms of currently used CHCs in the treatment of cholelithiasis and to describe the current situation of traditional Chinese medicine (TCM) use for cholelithiasis. Several commonly used CHCs were used to illustrate these issues. We found that the mechanisms underlying the CHC treatments rely on the amelioration of the biliary dynamics factors, maintenance and protection of the liver function, reduction of the cholesterol and bilirubin levels, and regulation of the inflammatory reactions. CHCs as treatments based on TCM can ameliorate the overall bodily function, thereby preventing the recurrence of cholelithiasis. Appropriate application of CHCs would be beneficial for patients and clinicians, although the safety and efficacy of CHCs need further verification.

## 1. Introduction

Cholelithiasis is a common biliary tract disease with high morbidity of approximately 10%–15% [1]. The most dominant symptom of cholelithiasis is severe abdominal pain accompanied with chills, fever, and jaundice recurrence; these symptoms substantially impact the quality of life (QOL) of the patients. Notably, only <20% of patients with cholelithiasis develop evident symptoms, whereas the remaining, particularly those with mild cholelithiasis, are typically asymptomatic [2]. However, the absence of symptoms does not indicate fitness. When the pathophysiological factors are not suppressed in asymptomatic patients, the disease may progress, resulting in serious consequences. Hence, cholelithiasis is a major public health concern that necessitates highly effective, feasible, and recurrence-preventing therapies. However, the recurrence of cholelithiasis is a crucial concern for the existing treatments, which is attributable to the complex pathogenic mechanisms of cholelithiasis. Cholelithiasis can be anatomically classified as gallstones, intrahepatic bile duct stones, and extrahepatic bile duct stones, and it can be chemically classified as cholesterol stones, pigmented stones, and mixed stones. Different types of cholelithiasis may exhibit different pathogenesis. Typically, the development of cholelithiasis is closely associated with impairments in the metabolism of bile. The most predominant impairments are abnormal bilirubin and lecithin levels. Ahmed et al. confirmed that serum bilirubin may bind with free metal ions, particularly calcium ions, thereby forming relevant bilirubinate [3]. Conversely, lecithin polymerizes the insoluble microparticles with bile acid during bile secretion [4]. These insoluble products can cause gallstone formation. Moreover, cholelithiasis in advanced stages is occasionally accompanied with complications that may affect the pathophysiology of cholelithiasis. For example, gallstones stimulate the gallbladder mucosa, leading to chronic inflammation and acute secondary infection of the gallbladder [5]. Intrahepatic bile duct stones are associated with the occurrence of intrahepatic cholangiocarcinoma [6]. These complicated diseases may further deteriorate the QOL and lead to a poor treatment outcome. Therefore, making an appropriate diagnosis and subsequent effective treatments during the early stages, particularly for asymptomatic patients, is crucial to improve the clinical outcome of patients with cholelithiasis.

Nowadays, both surgical and nonsurgical treatments are available for cholelithiasis. The surgical treatments, namely, lithotripsy and laparotomy, are rarely used for cholelithiasis treatment. Traditionally, laparoscopic cholecystectomy (LC) is the gold standard treatment for symptomatic gallstones. However, LC includes potential surgical risks, such as biliary bleeding, bile duct stricture, common-bile-duct recurrence stones, and postcholecystectomy syndrome, and it may also increase the incidence of colon cancer [7]. Endoscopic retrograde cholangiopancreatography (ERCP), which was first reported in 1974, has been used as a standard treatment for bile duct stones. Most bile duct stones (>90%) can be diagnosed and removed by any of the following techniques: ERCP, endoscopic sphincterotomy, or extraction balloon trawl [8]. However, ERCP has several limitations. It is an invasive surgery that may induce complications, such as cholecystitis and pancreatitis [9]. Treatment using ERCP is challenging in cases of large bile duct stones (>15 mm diameter) [10] and surgically altered anatomy [11]. Combining ERCP and LC in the same surgical session has been suggested to reduce possible conversions and complications [9]. Nakai et al. reported that, under endoscopic ultrasound guidance, enteroscopy-assisted ERCP is the potential technique for endoscopic management of bile duct stones in patients with surgically altered anatomy [11]. With the development of endoscopic technology, laparoscopic cholecystolithotomy with gallbladder preservation (LCGP) has been proposed by numerous clinicians because LCGP preserves gallbladder function, is minimally invasive, causes less surgical injury, and results in better QOL [12]. However, the efficacy of LCGP is controversial. The primary concerns toward LCGP are the efficacy of stone removal and its recurrence rate. In addition, the relatively strict indications of LCGP, such as gallbladder contraction function, gallbladder wall thickness, and gallstone characteristics (location, size, and number), limit the long-term use of LCGP. Meanwhile, the most commonly used medications for cholelithiasis are litholytic drugs, such as deoxycholic acid. However, the use of these medications is limited by expensive cost and noticeable adverse events, such as the development of hepatotoxicity and atherosclerosis [13].

Recurrence of gallstone formation is the major challenge for both clinicians and patients. None of the aforementioned treatments, including LC, ERCP, LCGP, surgical treatment, medications, and even complete gallbladder removal, can effectively prevent the recurrence of bile duct stones. Hence, several Chinese herbal compounds (CHCs) are considered for cholelithiasis treatment because they can effectively discharge gallstones [14] and prevent such recurrence [15–22]. Additionally, noninvasive traditional Chinese medicines (TCM) are easily accepted by asymptomatic patients who may potentially refuse surgical treatments. However, a systematic review in 2013 showed that the Chinese herbs such as Qingdan, Danshu, Paishi, and Rongdanpaishi capsules are not efficacious for cholelithiasis treatment. However, the studies included in this review are of poor quality, thereby limiting the worthiness of this published evidence [23]. Currently, several novel CHCs have been developed. The present narrative review aims to summarize the underlying mechanisms of currently used CHCs in the treatment of cholelithiasis and describe the current situation of TCM use for cholelithiasis.

## 2. Current Situation of TCM Use for Cholelithiasis

TCM has been used to treat cholelithiasis for centuries. The knowledge on TCM in relation to cholelithiasis was obtained by studying the autopsy and observing the discharged stones in ancient China, when modern diagnostic equipment was not yet available. All TCM therapies, including acupuncture and CHCs, were selected based on the TCM syndromes.

Acupuncture is often employed to relieve pain in patients. It is predominantly effective in alleviating biliary spasm and pain [24]. The common acupoints for treating cholelithiasis are the gallbladder, liver, and bladder meridians, such as Danshu (BL19), Riyue (GB24), and Qiuxu (GB40). However, studies indicating the benefit of acupuncture in discharging gallstones, attributed to its strengthening effect on the gallbladder contraction and bile secretion, are limited [25–27].

CHCs have been used for treating lithiasis, including cholelithiasis and kidney stones. Selection of the CHCs is based on the principles of syndrome differentiation treatment. In TCM, cholelithiasis is divided into the following five types according to the TCM syndromes: (1) Liver depression and qi stagnation (syndrome of stagnation of the liver and qi), (2) Wetness heat of liver and gallbladder (syndrome of dampness-heat of the liver and gallbladder), (3) Liver yin deficiency (liver yin deficiency syndrome), (4) Blood stasis stagnation (blood stasis syndrome), and (5) Heat-toxic stagnation. The newest guidelines for using CHCs are as follows: (1) good gallbladder function, (2) stone size < 10 mm in diameter, (3) absence of stricture at the bottom of the bile duct, and (4) applicability of CHCs for both extrahepatic and intrahepatic stones, as well as the residual stones following surgery [28]. Only few CHCs are approved by the China Food and Drug Administration (CFDA) for treating cholelithiasis. Thirteen CHCs are presented in Table 1. We included the CHCs used in the previous systematic review wherein their efficacies were refuted [23]. Interestingly,* Lysimachia christinae *is included in eight out of the thirteen CHCs. It is the only ingredient in Jin Qian Cao capsule. Hence,* L. christinae* plays a role in inducing the excretion of gallstones, as demonstrated in animal models [29]. Furthermore, n-butanol is the most bioactive fraction in the* L. christinae *extract; it exerts antioxidant effect and endothelial protection [30]. Moreover, Deng et al. suggested that* L. christinae *promotes bile secretion, reduces cholesterol levels in either cystic or hepatic bile, and decreases serum cholesterol level [29]. Although complex activities of the monomers in* L. christinae* remain poorly understood, the pharmacological effects of* L. christinae* need to be investigated to understand its underlying mechanisms in cholelithiasis treatment.

### 2.1. CHCs Contain L. christinae

#### 2.1.1. Changgen Antilithiasis Therapy (CALT)

**Figure 1:**
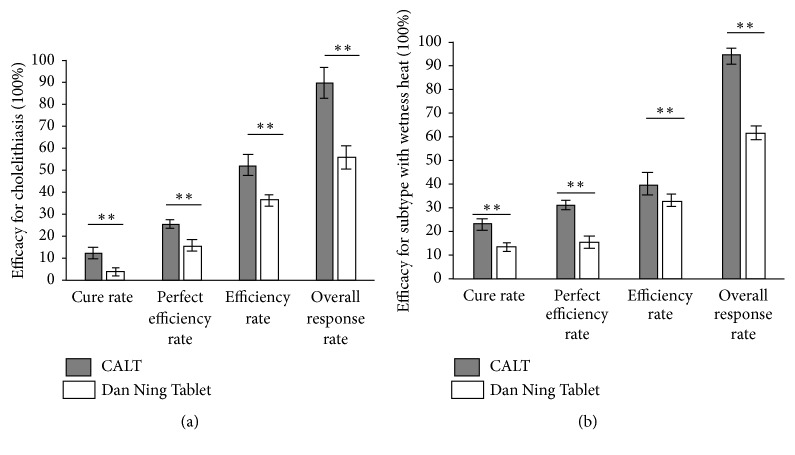
Efficacy of CALT in comparison with Dan Ning tablet. (a) CALT shows better efficacy in treating cholelithiasis than Dan Ning tablet. (b) CALT shows better efficacy in treating cholelithiasis of a subtype with witness heat than Dan Ning tablet. Data are presented as mean ± SD, *∗∗* indicates p < 0.01.

CALT is a CHC system comprising several formulas that have been used in treating cholelithiasis and kidney stones for 60 years. Jin Qian Dan Tong oral liquid, which is one of the CALT formulas, is approved by CFDA particularly for cholelithiasis treatment in China. The efficacy of CALT for cholelithiasis has been proved by several randomized controlled trials (RCTs) [14, 31, 32]. CALT promotes the discharge of gallstones and promptly relieves the pain [14, 31, 32]. Moreover, it is beneficial for the subtype with wetness heat [14, 31]. A clinical observation found that CALT promotes bile secretion [33]. Moreover, it ameliorates the biliary motility disorder in patients who underwent LC or ERCP [34]. Interestingly, CALT has shown better efficacy than a CHC without* L. christinae* (Dan Ning tablet) in treating cholelithiasis (Figure 1) [14]. Hence,* L. christinae* is a crucial ingredient of CHCs and warrants further investigations.

#### 2.1.2. Jin Dan Tablet (JDT)

Zhao et al. investigated the efficacy of JDT by comparing the combination of JDT and ursodeoxycholic acid with ursodeoxycholic acid alone. They observed that the JDT group exhibited a significantly higher efficiency rate than ursodeoxycholic acid alone (92.5% vs. 60.0%). Moreover, JDT relieved the adverse reactions associated with ursodeoxycholic acid, and the JDT group exhibited relatively lower occurrences of palpitation, blush, and loose stool than its counterpart [35].

#### 2.1.3. Pai Shi Li Dan Granules (PSLDG)

Pai Shi Li Dan granules may ameliorate postoperative complications affecting the gallbladder wall [36]. A 2-year follow-up study found that the recurrence rate of gallstones in Pai Shi Li Dan granules group was low, indicating that these granules might be effective in preventing postoperative recurrence of gallstones [22].

#### 2.1.4. Li Dan Pai Shi Tablet (LDPST)

Zhang et al. reported that Li Dan Pai Shi tablet could reduce residual gallstones in patients with intrahepatic bile duct stones. Moreover, it helps prevent the recurrence of gallstones [19].

#### 2.1.5. Dan Le Capsule (DLC)

Dan Le capsule is a common TCM medicine used for treating cholelithiasis. Its efficacy was verified by a clinical observation with 73 cases. It can relieve symptoms caused by cholelithiasis and has a gallbladder-stone-discharging effect [37].

#### 2.1.6. Dan Shi Li Tong Tablet (DSLTT)

He et al. conducted an RCT to verify the efficacy of Dan Shi Li Tong tablet. They found that the patients treated with such tablet achieved better cure rate (57.78% vs. 40.00%, p < 0.05) and total efficiency rate (95.56% vs. 73.33%, p < 0.05) than the control. This tablet can also ameliorate bilirubin metabolism and relieve the clinical symptoms [38].

### 2.2. CHCs without* L. christinae*

#### 2.2.1. Dan Qing Capsule (DQC)

Dan Qing capsule combined with ursodeoxycholic acid is used for treating cholelithiasis. Dan Qing capsule reduces the scores of TCM syndromes (lower score indicates better outcome), ameliorates gallbladder function, and relieves abdominal pain. Moreover, it prevents the recurrence of gallstones [17, 18].

#### 2.2.2. Dan Shi Tong Capsule (DSTC)

An RCT verified the efficacy of Dan Shi Tong capsule. A total of 60 patients with cholelithiasis were randomly allocated into treatment group (n = 30) and control group (n = 30). Study results revealed that the total efficiency rate of patients treated with Dan Shi Tong capsule was significantly higher than that of the control group (90% vs. 73.3%, p < 0.01). They concluded that Dan Shi Tong capsule can remarkably relieve the clinical symptoms of cholelithiasis. Additionally, it has a good stone-discharging effect, and no adverse effect was observed [39].

#### 2.2.3. Yi Dan Tablet (YDT)

One study has proven the efficacy of Yi Dan tablet in treating cholecystitis and cholelithiasis. The efficiency rate of this tablet on cholecystitis was >90% [40].

#### 2.2.4. Dan Ning Tablet (DNT)

Dan Ning tablet contributes to a reduced recurrence rate of gallstone formation following gallstone removal surgery (6.82% vs. 13.64%). The gallbladder contraction index in the treatment group was better than that in the control group. The underlying recurrence-preventing mechanism might involve the elimination of biliary inflammation by regulating the intestinal flora translocation [20, 21].

#### 2.2.5. Dan Yi Ning Tablet (DYNT)

Administering Dan Yi Ning tablet for cholelithiasis resulted in 76.56% stone removal rate, with 17.97% of patients achieving complete gallstone discharge. The mechanisms might be associated with the improvement of the biliary dynamics factors [41].

#### 2.2.6. Dan Shi Qing Tablet (DSQT)

Bao et al. designed an RCT with 45 cases to investigate the efficacy of Dan Shi Qing tablet (Control group [ursodeoxycholic acid] vs. Treatment group [ursodeoxycholic acid + Dan Shi Qing tablet]). After a 2-year follow-up, they found that Dan Shi Qing tablet had a significantly lower recurrence rate than the control (16% vs. 25% in the control group, p < 0.01). Hence, this tablet can reduce the postoperative recurrence rate of gallstones [42].

Aside from the abovementioned CHCs, many other CHCs are also commonly used for treating cholelithiasis. However, considering that no clinical trials have been reported to verify the efficacy of these CHCs (such as Jie Shi Qing capsule, Dan Shi tablet, Dan Shu Soft capsule, Shu Dan capsule, Jin Jia Pai Shi Capsule, Jin Qian Cao Capsule, Dan Kang capsule, and Li Dan Shi granule) or these medicines are not mainly used to treat cholelithiasis (such as Li Dan Xiao Yan Tablet, which is mainly used for cholecystitis), we did not introduce them in detail in accordance with the aim of this study.

## 3. Potential Mechanisms Underlying the CHC Treatments

### 3.1. Amelioration of the Biliary Dynamics Factors

Changes in the biliary fluid dynamics play a crucial role in the development of gallstones [43]. Filling and emptying of the gallbladder are the results of the synergistic motility of the gallbladder, gallbladder duct, and the sphincter of Oddi. If the dynamics of the bile duct and/or gallbladder is disrupted, it may change the flow direction or produce swirls or eddies that may result in cholestasis and promote the development of cholelithiasis. As mentioned, CALT ameliorates postoperative biliary motility disorder. Indices such as the common-bile-duct pressure and the basal pressure in the sphincter of Oddi were significantly reduced by CALT administration [34]. This phenomenon might be a potential mechanism underlying the recurrence prevention. Sheng et al. reported that the 2-week administration of Dachengqi decoction, which comprises* Rheum officinale*, glauber,* Fructus aurantii immaturus*, and* Magnolia officinalis*, significantly enhanced the gallbladder emptying index, indicating that the gallbladder contraction had strengthened [44]. Fang et al. revealed that emodin may ameliorate the dysfunction of gallbladder contraction and relieve cholestasis by enhancing the gallbladder contractibility. The regulation of the plasma cholecystokinin and calcium ion in the gallbladder cells may play a role in the underlying mechanisms [45]. Therefore, CHCs can strengthen gallbladder contraction [20, 21], promote bile secretion (Section 3.2), and easily discharge gallstones.

### 3.2. Maintenance and Protection of the Liver Function

Bile is mainly produced by the liver. When the liver is impaired, cholelithiasis may occur. Certain pathological conditions cause the liver to produce excessive cholesterol (Section 3.3) or the liver cells to synthesize superfluous bilirubin (Section 3.4), thereby resulting in the development of gallstones [46]. Reduced bile secretion causes pachycholia, possibly related to the development of gallstones. Meanwhile, the progression of cholelithiasis would adversely affect the liver function and damage the liver cells, thereby forming a vicious circle. Another example is the relationship between liver fat metabolism and cholelithiasis. An imbalance in liver fat metabolism may lead to the development of cholelithiasis [47]. Repairing liver injury and restoring the balance of liver fat metabolism would allow the recovery of cholelithiasis or prevention of gallstone formation [48]. Therefore, cholelithiasis can be ameliorated by improving the liver function. Meanwhile, CALT promotes bile secretion [33], possibly a result of an ameliorated liver function. Furthermore, according to the theories of TCM, the maintenance and protection of the liver function play a crucial role in the mechanisms of CHCs. One CHC not containing* L. christinae*, i.e., the Dan Ning tablet, exerts protective effects on an acutely injured liver. Administration of this tablet reduces the levels of serum alanine aminotransferase, aspartate aminotransferase, alkaline phosphatase, *γ*-glutamyltranspeptidase, total bilirubin, direct bilirubin, and total bile acid, as well as the hepatic myeloperoxidase, glutathione S-transferase activities, and contents of glutathione and liver lipid peroxide; in contrast, the activities of superoxide dismutase, glutathione peroxidase, and catalase were enhanced. Hence, the attenuation of the oxidative stress may be the underlying mechanism of cholelithiasis treatment using Dan Ning tablet [49].

### 3.3. Reduction of the Serum Cholesterol Level

Cholesterol is an essential constituent of bile. It is dissolved in the bile. When the ratio of cholesterol/phospholipids is >1, the existing micelles are insufficient to dissolve the whole cholesterol content. Consequently, the excessive cholesterol begins to crystallize and nucleate, indicating the mechanism of cholesterol stone formation [50]. Furthermore, the reduction of serum cholesterol level might be a result of an ameliorated liver function (Section 3.2). However, the mechanisms are complicated and multifold. Liu reported that schaftoside, which is a bioactive compound of* L. christinae,* reduces serum cholesterol level; this decrement is vital for preventing the development of cholesterol gallstones. These effects are obtained by activating the receptors of ileal liver X receptor *α* and hepatic farnesoid X receptor [51]. Another study regarding Lidan granules (*Oriental wormwood, Hawthorn fruit, Rice sprout, Germinated barley, Green orange peel, Tangerine peel, Medicated leaves, Cyperus tuber, Radish seed, Caulis perillae, Turmeric root, Rhubarb, Pinellia tube, and Chinese honeylocust fruit*) demonstrated that reducing cholesterol level and increasing bile phospholipid levels by downregulating the expression of inflammatory cytokines are the underlying mechanisms involved in preventing cholelithiasis [52].

### 3.4. Reduction of the Bilirubin Level

Bilirubin is the main pigment in bile. In certain pathological states, the bilirubin level in the bile may be enhanced and contribute to the development of bilirubin gallstones. The risk of developing gallstones is closely associated with a high bilirubin level [53]. In contrast, relieving the pathogenic factor that enhances the bilirubin (such as ameliorating the liver function) can improve and prevent bilirubin gallstones. A previous study reported the efficacy of Jinshisan in treating gallstones and discovered that this CHC is effective in preventing bile stone formation. The efficacy of Jinshisan treatment is highly associated with a reduction of bilirubin level in the bile [54].

### 3.5. Regulation of the Inflammatory Reactions

The roles of inflammatory factors in the development of gallstones are multifold and complicated. Briefly, inflammatory reactions may damage liver function, disturb biliary fluid dynamics, and cause an imbalance in bile production and secretion, thereby resulting in cholelithiasis [55]. Dan Ning tablet is efficacious in ameliorating cholelithiasis by attenuating the oxidative stress [49]. Liu et al. reported that CHCs promote bile excretion, regulate bile components, and protect the gallbladder mucosal epithelial cells. The underlying mechanisms include the suppression of cyclooxygenase-2 and tumor necrosis factor-*α* expression via the nuclear factor-kappa B signaling pathway [56]. Another study found that CHCs may regulate the expression of IL-6 and EGR-1 in the hepatic tissue of a rabbit cholelithiasis model. Furthermore, chronic liver injury due to cholelithiasis was treated, owing to the recovery of the liver function and protection of normal hepatic cells [57]. Inflammatory factors, such as cytokines, and the inflammatory signaling pathway are crucial in the therapeutic mechanisms of CHCs in gallstones. Further investigations are expected to uncover the detailed inflammatory processes regarding the effects of CHCs.

## 4. Concluding Remarks

The present review summarized the underlying mechanisms of commonly used CHCs for cholelithiasis treatment. The CHCs included in the present review are frequently used clinically for treating cholelithiasis in China. Considering that we require the latest comprehensive information based on the contents of available studies, an updated systematic review is needed in the future.

**Figure 2:**
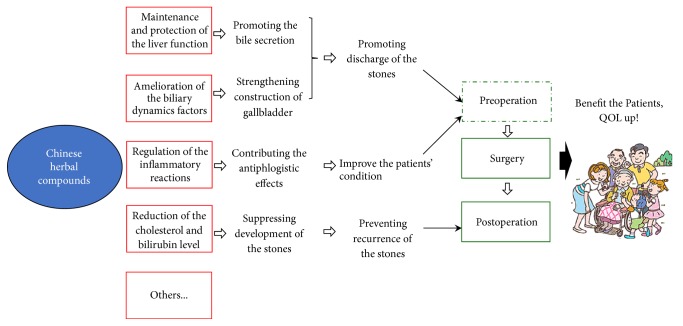
Underlying mechanisms of CHCs involved in the clinical strategy of cholelithiasis.

The commonly used therapeutics, regardless of being surgical or medical, cannot circumvent the recurrence of gallstones. However, the CHCs can maintain and protect the liver function, improve biliary fluid dynamics, and regulate the inflammatory reaction. Theoretically, CHCs can amend the lithogenic environment either pre- or postoperatively. Hence, CHCs should prevent the recurrence of gallstones, and several studies have supported this statement [15–22]. Therefore, more well-designed RCTs are needed to provide convincing evidence regarding the effectiveness of CHCs in treating cholelithiasis. Once the effects of preventing the recurrence of cholelithiasis are rigorously verified, these CHCs can be considered as a useful adjuvant therapy for postoperative patients. CHCs can ameliorate the patients' condition perioperatively and prevent them from experiencing the recurrence of gallstones postoperatively (Figure 2).

Notably, none of the RCTs that verified the efficacy of the involved CHCs reported the adverse events. This finding is similar to that of our previous study [58–60]. Indeed, the lack of report regarding adverse events and follow-up has been a dominant problem in the TCM research [58, 59]. Several Chinese patients and TCM researchers traditionally believe that TCM therapies have no or few adverse effects [23]. However, this view is far from the truth. Our previous study reported that acupuncture cannot be regarded as a completely noninvasive therapy [61]. Recently, adverse events of CHCs have also been documented, thereby gaining considerable attention worldwide. Furthermore, the relationship between aristolochic acids and hepatocellular carcinomas has been recognized [62]. A current study found that the abuse of CHCs has been the leading cause of drug-induced liver injury (26.81%) in China [63]. Regarding CHCs for treating cholelithiasis, no report is available that systematically introduces the adverse events/side effects of these medicines. However, some components of Dan Ning tablets (anthraquinone) potentially induce melanosis coli (MC) [64]. In addition, 2 out of 48 patients with MC had a history of Dan Ning tablet administration [65]. Nevertheless, the relationship between Dan Ning tablets and MC needs further investigation. However, all of these cases suggest that the adverse events of CHCs for cholelithiasis cannot be disregarded and should be seriously considered in future TCM studies. Measures such as spreading awareness concerning the adverse events of TCM in both the TCM clinicians and the general population along with establishing and improving the reporting systems are helpful to improve this problem [66]. Another important drawback of the involved studies was the lack of blinding and allocation concealment. This is a systematic problem involved in the TCM research [61]. The characteristics of TCM are “concept of wholism (holistic concept)” and “syndrome differentiation treatment (pattern differentiation).” These characteristics may cause blinding (especially the double blinding) and allocation concealment, which are rather difficult to be practiced in a TCM trial (the clinician must perform the individual and dynamic treatment protocol for each patent). Adoption of objective indices to prevent the results from being changed by the subjectivity from different observers (observation bias) is a good solution for this issue [61]. Hence, objectification (for both diagnosis tool and treatment) may be potentially applied in future TCM studies.

Cumulatively, TCM is characterized by the concept of wholism (holistic concept) and syndrome differentiation treatment (pattern differentiation). CHCs as treatments based on TCM can ameliorate the overall bodily function, thereby preventing the recurrence of illness. Appropriate application of CHCs would be beneficial for patients and clinicians, although their safety and efficacy need further verification.

## Figures and Tables

**Table 1:** Commonly used CHCs approved by CFDA.

CHC Name	Ingredients
Changgen Anti-Lithiasis Therapy (CALT, former name: Jin Qian Dan Tong Oral Liquid)	*Herba Lysimachia, Lysimachia christinae, Oriental wormwood, Polygonum cuspidatum, Radix bupleuri, Dandelion, Cyperus tuber, Root of red-rooted salvia, Semen cassiae torae, Smoked plum*

Jin Dan Tablet	*Radix gentianae, Lysimachia christinae, Polygonum cuspidatum, Pig's bile*

Pai Shi Li Dan Granules	*Lysimachia christinae, Oriental wormwood, Radix bupleuri, Radix gentianae, Radix paeoniae rubrathe (unpeeled) root of common peony, Radix curcumae, Cattail pollen, Excrementum pteropi, Rheum officinale, Glauber*

Li Dan Pai Shi Tablet	*Lysimachia christinae, Oriental wormwood, Scutellaria baicalensis, Radices saussureae, Radix curcumae, Rheum officinale, Gglauber, Areca catechu, Fructus aurantii immaturus, Magnolia officinalis*

Dan Le Capsule	*Pig's bile, Pericarpium citri reticulatae, hawthorn, curcuma aromatica, Herba Lysimachia*

Dan Shi Li Tong Tablet	*Alum, Glauber, Radix curcumae, Rhizoma sparganii,Pig's bile, Lysimachia christinae, Pericarpium citri reticulatae, Frankincense,myrrh, Rheum officinale, Radix liquiritiae*

Jin Jia Pai Shi Capsule	*Rhizoma sparganii, myrrh, Radix paeoniae rubrathe (unpeeled) root of common peony, Peach kernel, Chinese honeylocust fruit, Angelica dahurica, Fructus aurantii immaturus, Curcuma zedoary, Pericarpium citri reticulatae viride, Frankincense, Semen coicis, Radices cyathulae, Plantago seed, Magnolia officinalis, Pangolin, Lysimachia christinae*

Jin Qian Cao Capsule	*Lysimachia christinae*

Dan Qing Capsule	*Saxifraga stolonifera, Phoenix-tail fern, Rheum officinale, Oxgall*

Dan Shi Tong Capsule	*Dandelion, Radix curcumae, Oriental wormwood, Lycopodium mongolicum, linearstripe rabdosia, Rheum officinale, fructus aurantia, Radix bupleuri, Scutellaria baicalensis, Goose gall*

Yi Dan Tablet	*Radix curcumae, Honeysuckle, Alum, Liquorice, Glauber, Talc, Radix scrophulariae*

Dan Yi Ning Tablet	*Pig's bile, Plum root*

Dan Shi Qing Tablet	*Alum, Oxgall, Sheep bile, hawthorn, Radix curcumae, Rheum officinale,Glauber, clematis chinensis, Endothelium corneum gigeriae galli, Glauber*

## Data Availability

The data used to support the findings of this study are included within the article.
